# Phase Noise of SAW Delay Line Magnetic Field Sensors

**DOI:** 10.3390/s21165631

**Published:** 2021-08-21

**Authors:** Phillip Durdaut, Cai Müller, Anne Kittmann, Viktor Schell, Andreas Bahr, Eckhard Quandt, Reinhard Knöchel, Michael Höft, Jeffrey McCord

**Affiliations:** 1 Microwave Engineering, Institute of Electrical Engineering and Information Technology, Faculty of Engineering, Kiel University, Kaiserstr. 2, 24143 Kiel, Germany; rk@tf.uni-kiel.de (R.K.); mh@tf.uni-kiel.de (M.H.); 2 Nanoscale Magnetic Materials and Magnetic Domains, Institute for Materials Science, Faculty of Engineering, Kiel University, Kaiserstr. 2, 24143 Kiel, Germany; camu@tf.uni-kiel.de (C.M.); jmc@tf.uni-kiel.de (J.M.); 3 Inorganic Functional Materials, Institute for Materials Science, Faculty of Engineering, Kiel University, Kaiserstr. 2, 24143 Kiel, Germany; anki@tf.uni-kiel.de (A.K.); visc@tf.uni-kiel.de (V.S.); eq@tf.uni-kiel.de (E.Q.); 4 Sensor System Electronics, Institute of Electrical Engineering and Information Technology, Faculty of Engineering, Kiel University, Kaiserstr. 2, 24143 Kiel, Germany; ab@tf.uni-kiel.de

**Keywords:** Barkhausen noise, delay line sensor, Flicker noise, Kerr microscopy, magnetic domain networks, magnetic field sensor, magnetic noise, magnetoelastic delta-E effect, phase noise, surface acoustic wave

## Abstract

Surface acoustic wave (SAW) sensors for the detection of magnetic fields are currently being studied scientifically in many ways, especially since both their sensitivity as well as their detectivity could be significantly improved by the utilization of shear horizontal surface acoustic waves, i.e., Love waves, instead of Rayleigh waves. By now, low-frequency limits of detection (LOD) below 100 pT/$\sqrt{\mathrm{Hz}}$ can be achieved. However, the LOD can only be further improved by gaining a deep understanding of the existing sensor-intrinsic noise sources and their impact on the sensor’s overall performance. This paper reports on a comprehensive study of the inherent noise of SAW delay line magnetic field sensors. In addition to the noise, however, the sensitivity is of importance, since both quantities are equally important for the LOD. Following the necessary explanations of the electrical and magnetic sensor properties, a further focus is on the losses within the sensor, since these are closely linked to the noise. The considered parameters are in particular the ambient magnetic bias field and the input power of the sensor. Depending on the sensor’s operating point, various noise mechanisms contribute to $f^{0}$ white phase noise, $f^{-1}$ flicker phase noise, and $f^{-2}$ random walk of phase. Flicker phase noise due to magnetic hysteresis losses, i.e. random fluctuations of the magnetization, is usually dominant under typical operating conditions. Noise characteristics are related to the overall magnetic and magnetic domain behavior. Both calculations and measurements show that the LOD cannot be further improved by increasing the sensitivity. Instead, the losses occurring in the magnetic material need to be decreased.

## 1. Introduction

Since the invention of the interdigital transducer (IDT) in 1965, surface acoustic waves (SAW) can be excited very efficiently on piezoelectric materials [1]. Due to their small size, low cost, and high reproducibility, SAW filters have taken on a key role in modern consumer and communication systems [2]. The same advantageous properties make SAW technology attractive for sensor applications [3,4].

Utilizing the inverse piezoelectric effect, a SAW is excited by applying an electrical voltage on an (input) IDT that is patterned on a piezoelectric material. The mechanical wave propagates perpendicular to the direction of the IDT in both directions on the surface of the piezoelectric substrate ([5], p. 139). For sensing applications the substrate’s surface is frequently coated with an additional layer which reacts to changes of the physical quantity to measure and, in turn, alters the propagating wave in its amplitude and in its velocity. For the detection of externally affected wave properties such a device can be equipped with an additional (output) IDT, thus forming a so-called delay line structure. Due to the reciprocity of IDTs and via the direct piezoelectric effect the mechanical wave is then converted back into an electrical signal.

The operating principle of SAW delay line magnetic field sensors [6] is based on the magnetoelastic ΔE effect. It leads to changes of the Young’s modulus *E* and the related shear modulus *G*, respectively, of an additional magnetostrictive layer as a function of the material’s magnetization *M*, i.e., of an ambient magnetic flux density $B=\mu_{0}H$ ($\mu_{0}$ and *H* denote the vacuum permeability and the magnetic field strength). Due to the relation between the mechanical property *G* and the wave’s propagation velocity *v* [7] the phase φ of a shear wave magnetic field sensor’s output signal is a function of the magnetic flux density *B* with the phase sensitivity

(1) $$S_{\mathrm{PM}}=\frac{\partial \varphi }{\partial B}=\frac{\chi }{\mu_{0}}\;\cdot \;\frac{\partial G}{\partial M}\;\cdot \;\frac{\partial v}{\partial G}\;\cdot \;\frac{\partial \varphi }{\partial v}$$

where χ denotes the magnetic susceptibility χ=∂M/∂H.

The first magnetoelastic SAW delay line devices were presented in the 1970s for the possible application as magnetically tunable phase shifters [8,9], e.g., in frequency-tunable oscillators [10]. The use of soft magnetic materials such as iron-boron (FeB) instead of hard magnetic nickel (Ni) leads to lower ambient magnetic flux densities *B* required for achieving a significant phase shift [11]. To further increase the effect or the maximum phase shift, respectively, the thickness of the magnetic layer was increased and various magnetic alloys were applied [12,13,14,15,16,17,18,19]. In 1992, Yokokawa et al. demonstrated that the magnetically induced phase shift can be significantly increased if shear horizontal surface acoustic waves, i.e., Love waves, instead of Rayleigh waves are excited [20].

The first magnetoelastic delay line magnetic field sensor capable of detecting changes of 1 μT was presented in 1987 [21]. It was not until 30 years later that magnetically coated delay lines were again operated as sensors [22] with an achieved detection limit of 140 nT [23]. The first sensor explicitly exploiting the high sensitivity of Love waves was presented in 2018 reaching a limit of detection (LOD) of 250 pT/$\sqrt{\mathrm{Hz}}$ at a frequency of 10 Hz [24]. Besides utilizing higher Love modes in the Gigahertz regime [25] also resonant surface acoustic Love wave magnetic field sensors have been introduced in the last three years [26,27,28]. Meanwhile, Love wave delay line sensors reach limits of detection as low as 70 pT/$\sqrt{\mathrm{Hz}}$ at a frequency of 10 Hz [29]. Thus, such sensors are already significantly more detective than state-of-the-art Hall effect sensors with typical limits of detection around 1 μT/$\sqrt{\mathrm{Hz}}$ at a frequency of 10 Hz [30]. Currently, the LOD of SAW magnetic field sensors is most comparable with magnetoresistive sensors with values around 100 pT/$\sqrt{\mathrm{Hz}}$ at 10 Hz [31,32]. However, giant magnetoimpedance [33] and fluxgate sensors [34], for example, still achieve significantly better low-frequency values around or even below 10 pT/$\sqrt{\mathrm{Hz}}$.

Apart from the fact that even values on measured limits of detection are rarely given, no detailed results on the noise behavior of magnetoelastic SAW delay line sensors have been reported so far. In a recent study on the required readout electronics for the operation of such delay line sensors it was found that magnetostrictively coated SAW devices can exhibit significantly increased noise compared to bare devices without magnetic material [35]. In previous studies on the effective noise of other types of thin-film magnetic field sensors, direct links to the magnetic domain behavior have been determined [36]. Therefore, the LOD of SAW magnetic field sensors based on magnetic thin films can only be further improved by gaining a deep understanding of the existing sensor-intrinsic noise sources with an emphasis on the magnetic domain behavior and its impact on the sensor’s overall performance.

This paper is organized as follows: Section 2 introduces the SAW magnetic field sensor under investigation and discusses its electrical and magnetic behavior. The comprehensive analysis of the intrinsic phase noise of SAW delay line magnetic field sensors is divided into two parts. First, in Section 3, phase noise occurring in magnetically saturated devices as well as in devices without any sensitive coating is discussed. Secondly, additional phase noise phenomena due to the magnetostrictive layer are presented and analyzed in Section 4. This article finishes with a summary of the findings in Section 5.

## 2. SAW Sensor

A delay line is formed using two split-finger IDT electrodes with 25 finger pairs, a periodicity, i.e., an acoustic wavelength, of λ=28 μm, and a finger width of 3.5 μm with an IDT center-to-center length of L=4.64 mm. An SiO${}_{2}$ layer with a thickness of 4.5 μm deposited on top of the IDTs and the delay line acts as a guiding layer for the surface acoustic Love wave. A magnetostrictive material (Fe${}_{90}$Co${}_{10}$)${}_{78}$Si${}_{12}$B${}_{10}$ with a thickness of z=200 nm and a length of 3.8 mm is magnetron sputter-deposited on top of the guiding layer and between the IDTs. During deposition, for maximizing the sensor’s magnetic sensitivity, a magnetic field is applied along the direction of the delay line to saturate the magnetic film and to introduce an easy axis of magnetization [29].

Further details about the fabrication can be found in [37]. The sensor mainly discussed in this paper has already been used in a previous study with a focus on the electrical readout systems which also contains a photography of the sensor [35].

### 2.1. Electrical Properties

For the electrical characterization, the two-port scattering parameters $s_{ij}\;\left(i,j\in \left\{1,2\right\}\right)$ of the sensor are measured with a calibrated vector network analyzer *E8361A* from *Agilent Technologies* at a signal power of $P_{0}=0\;\mathrm{dBm}$. In order to counteract additional magnetic influences (will be discussed further below), the magnetostrictive layer is magnetically saturated with a permanent magnet ($B=B_{\mathrm{sat}}\approx 10\;\mathrm{mT}$) perpendicular to the wave propagation direction, i.e., along the magnetic hard axis. To also minimize mismatch losses due to reflections at the electrical-acoustical interfaces, an individual impedance matching to the system impedance of $Z_{0}=50\;\Omega$ was carried out using discrete inductors and capacitors prior to all measurements. In addition, to suppress significant signal-dropping in the transmission characteristics due to interference of electrical crosstalk, the delay line is connected symmetrically utilizing a balun (*ATB2012-50011* from *TDK*) at each port.

**Figure 1:**
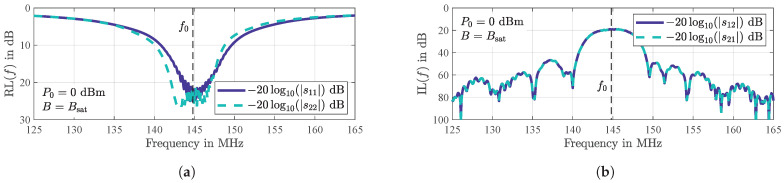
Measured two-port scattering parameters of the SAW sensor yielding a return loss (RL) better than 20 dB at each port (**a**) and an insertion loss (IL) with a value of 20 dB (**b**), both at the synchronous frequency of $f_{0}$ = 144.8 MHz. During the measurements, performed for an input power of $P_{0}$ = 0 dBm, the sensor has been magnetically saturated at $B=B_{\mathrm{sat}}\approx$ 10 mT in order to avoid additional magnetic influences (which will be discussed below).

Values for the return loss of $\mathrm{RL}\left(f_{0}\right)=-20\log_{10}\left(\right|s_{ii}\left(f_{0}\right)\left|\right)\;\mathrm{dB}>20\;\mathrm{dB}$(i∈{1,2}) are achieved at the sensor’s synchronous frequency of $f_{0}=144.8\;\mathrm{MHz}$ at each port (Figure 1a). The exactly measured values correspond with an overall mismatch loss ([38], pp. 64–65)

(2) $$\begin{array}{c} \mathrm{ML}\left(f\right)=10\log_{10}\left(\frac{1}{1-|s_{11}{\left(f\right)|}^{2}}\;\cdot \;\frac{1}{1-|s_{22}{\left(f\right)|}^{2}}\right)\mathrm{dB} \end{array}$$

as low as $\mathrm{ML}(f_{0})=0.04\;\mathrm{dB}$ which is negligible for the sensor under investigation. Thus, the measured insertion loss (Figure 1b) at $f_{0}$ with a typical value for Love wave delay lines [39] of $\mathrm{IL}\left(f_{0}\right)=-20\log_{10}\left(\right|s_{21}\left(f_{0}\right)\left|\right)\;\mathrm{dB}=20\;\mathrm{dB}$ is virtually solely determined by the SAW device itself.

### 2.2. Magnetic Properties

**Figure 2:**
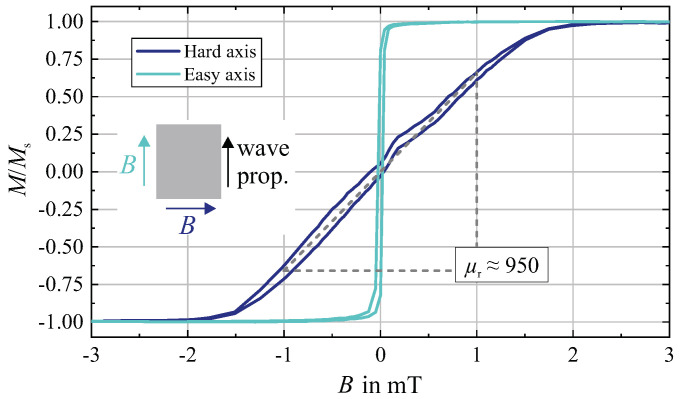
Magnetization loops along (easy axis) and perpendicular (hard axis) to the direction of wave propagation of the SAW device measured by magnetooptical magnetometry. The relative permeability perpendicular to the direction of wave propagation is around $\mu_{r}\approx 950$. The magnetic film geometry and the measurement directions are sketched.

For the magnetic characterization, magnetooptical Kerr effect (MOKE) magnetometry and magnetic domain observations were applied using a home-build large view MOKE setup [40,41] with telecentric optics. The magnetization loop measured along and perpendicular to the device dimensions shown in Figure 2 displays a well-defined soft magnetic uniaxial anisotropy behavior. With the saturation polarization $B_{s}=1.5\;T$ [42] a uniaxial anisotropy constant of $K_{u}\approx 960\;J/m^{3}$ is obtained. This corresponds to a relative permeability of $\mu_{r,\mathrm{ha}}\approx 950$ along the magnetic hard axis. The easy axis maximum permeability, governed by magnetic domain wall motion, is significantly higher and in the order of $\mu_{\mathrm{rmax},\mathrm{ea}}\approx 10,000$.

**Figure 3:**
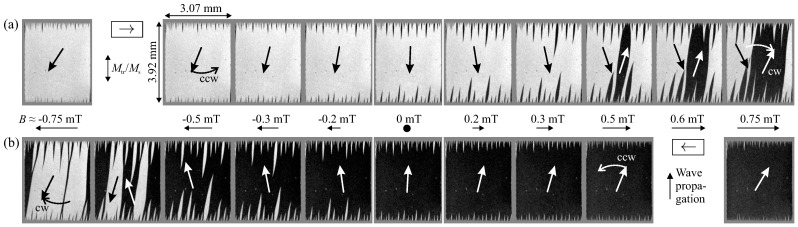
Magnetic domain evolution for increasing (**a**, →) and decreasing (**b**, ←) direction of external field *B*. Magnetic field values are indicated. The corresponding basic alignment of magnetization inside the magnetic film is sketched. The magnetooptical sensitivity is transverse to the applied magnetic field direction.

Relevant insight into the actual magnetization behavior is obtained from the magnetic domain behavior. A comparison of the magnetic domain behavior for ascending and descending loop branches perpendicular to the direction of wave propagation is shown in Figure 3. Two important points become obvious in the domain arrangement. First, the magnetic domain behavior is asymmetric. Different magnetic domain characteristics are found for the ascending and descending loop for a given magnetic field. Coming from magnetic saturation spike domains develop at the edges and magnetization rotation takes place in the center of the magnetic layer structure. The sign of initial magnetization rotation does not depend on the sign of applied saturation field *B*, it is counterclockwise (ccw). After remanence, the spike domains grow and further on penetrate the whole sample, forming large magnetic domains. The switched domains then rotate clockwise (cw) with further increase of field magnitude. Secondly, the spike domains as well as the central domain walls are slightly tilted with respect to the dimension of the device. Both findings indicate a slightly tilted magnetic anisotropy axis. Consequently, the magnetization firstly rotates towards the preferential anisotropy axis, counterclockwise for ascending and descending external field variations. Due to the resulting symmetry breaking, after reversing the magnetic field, the magnetization reversal from one to the now favored axis of anisotropy takes place by domain wall motion.

**Figure 4:**
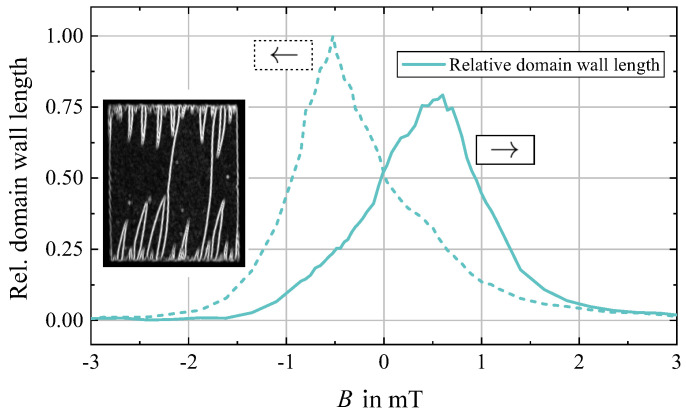
Relative magnetic domain wall evolution for increasing (→) and decreasing (←) direction of external field *B* obtained via edge detection from the magnetooptical micrographs. The intensity from the domain wall contrast obtained by the edge detection operation is interpreted as a value related to the magnetic domain wall length. An example on an edge detection filtered intensity analyzed image is shown.

To obtain a measure for the asymmetric magnetic domain and domain wall behavior, a simple Sobel filter implemented in the image processor ImageJ [43] is used for estimating the relative magnetic domain wall length with variation of *B*, the results of which are displayed in Figure 4. The development of domain walls displays a strong hysteresis with a relatively monotonous increase and then decrease in domain wall density. The domain wall density peaks around B≈∓0.5 mT for the reversed magnetic field application. Recapping, the magnetization process is asymmetric and reversing in characteristics with reversing magnetic field history, which is directly reflected in the magnetic domain wall density variation with field.

### 2.3. Electrically Induced Changes of the Magnetization Behavior

**Figure 5:**
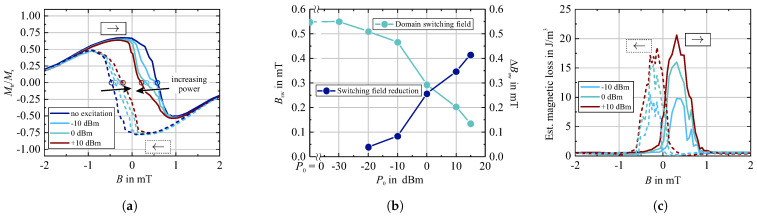
Exemplary transverse sensitivity magnetization loops for different values of electrical input power $P_{0}$ (**a**) and corresponding magnetic domain switching fields $B_{\mathrm{sw}}$ and magnetic switching field reduction $\Delta B_{\mathrm{sw}}$ with $P_{0}$ (**b**) derived from the transverse loops. The magnetic energy transfer (**c**) is estimated taking into account (**a**) and the easy axis magnetization characteristics (Figure 2). A static easy axis hysteresis loss of $B_{\mathrm{dw}}\approx$ 60 J/m^3^ is estimated from the easy axis loop. The varying electrical input power $P_{0}$ was set at or close to the synchronous frequency $f_{0}$ = 144.8 MHz of the device.

A quantitative measure of the magnetic domain switching behavior with varying electrical input power $P_{0}$ is obtained from an analysis of the magnetization behavior by MOKE transverse magnetometry [44] with the MO sensitivity aligned perpendicular to the magnetic field excitation. Exemplary transverse loop data on the switching behavior is displayed in Figure 5a.

No change in the regular magnetization loops with an increase of input power $P_{0}$ was found. Starting from negative values of *B*, the transverse magnetization component increases corresponding to a dominating ccw rotation of magnetization for the given MOKE settings (dark contrast in Figure 3, $M_{\mathrm{tr}}/M_{s}>0$). After remanence, the transverse magnetization component decreases due to the growth of reversed magnetized magnetic domains. The domain switching field $B_{\mathrm{sw}}$, with the same fraction of upward and downward magnetized domains, we define at the field with $M_{\mathrm{tr}}/M_{s}=0$ (note that these values are not equal to the coercive fields). The positions of $B_{\mathrm{sw}}$ are indicated in Figure 5a. With further reversing *B* ($M_{\mathrm{tr}}/M_{s}<0$) the sense of magnetization rotation inverts to cw rotation, again confirming the slightly tilted magnetic anisotropy axis. The switching process is accompanied by irregular stepwise change in the transverse magnetization, corresponding to domain wall or Barkhausen jumps. With the application of an electrical input power of $P_{0}$ the general magnetization behavior remains unchanged. Yet, the domain switching field decreases with increasing $P_{0}$. The overall decrease of $B_{\mathrm{sw}}$ with $P_{0}$ and the directly related reduction of the switching field $\Delta B_{\mathrm{sw}}$ is displayed in Figure 5b. Even for small levels of input power ($P_{0}=-20\;\mathrm{dBm}$) a measurable influence on the magnetic domain walls depinning fields is visible in the data. This effect on the magnetic domains, respectively magnetic domain walls we interpret as an energy transfer from the surface acoustic waves to the magnetic domain walls.

An estimation of the energy transfer with *B* is not straightforward but comparing the difference in the transverse magnetization loops relative to the zero-input state should give a rough approximation of the energy transferred to the magnetic domain walls. The difference $\Delta M_{\mathrm{tr}}/M_{s}$ is then compared to the hysteretic energy loss of the easy axis magnetization loop (∮HdB) where the overall magnetization response from negative to positive saturation is $2\;\cdot \;M_{\mathrm{tr}}/M_{s}$. This process is as well characterized by magnetic domain wall motion. Assuming an idealized square easy axis loop the hysteric loss $P_{\mathrm{dw}}$ is then defined by

(3) $$P_{\mathrm{dw}}=\frac{1}{2}\Delta M_{\mathrm{tr}}/M_{s}\;\cdot \;\oint H_{\mathrm{ea}}\;dB_{\mathrm{ea}}.$$

The corresponding field dependency is shown in Figure 5c. For the easy axis magnetization behavior the relevant energy densities are much smaller than the uniaxial anisotropy density. The estimated energy transfer peaks at the domain stability field, respectively, the domain switching field, as well as with the maximum in domain wall length (compare to Figure 2). Yet, the regime of relevant energy transfer is reduced and more asymmetric, respectively, enhanced in the domain switching regime. The linked relation of the magnetization response on the electrical properties is discussed next.

### 2.4. Magnetically Induced Changes of the Electrical Properties

**Figure 6:**
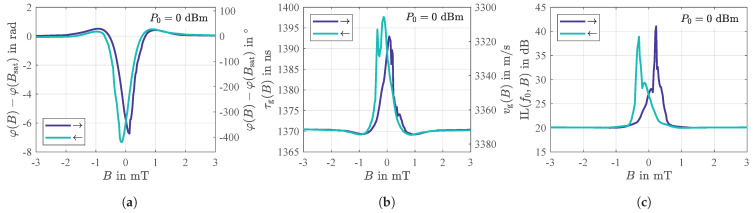
Phase response (**a**), group delay and group velocity (**b**), and insertion loss (**c**) of the SAW delay line sensor obtained from a series of measurements of the two-port scattering parameters for various static magnetic flux densities *B*. The results of all three sub-figures are based on data from the same series of measurements which was performed for an input power of the sensor of $P_{0}$ = 0 dBm and at or around, respectively, its synchronous frequency of $f_{0}$ = 144.8 MHz.

Characteristic magnetic influences on the SAW delay line sensor’s electrical behavior are extracted from a series of measurements of the sensor’s two-port scattering parameters as already described above but additionally for various ambient static magnetic flux densities *B*. The results are depicted in Figure 6.

The static magnetic fields are generated by means of a programmable current source *B2962A* from *Keysight* and a solenoid [45]. The sensor and the surrounding solenoid are placed inside an ultra high magnetic field shielding mu-metal cylinder *ZG1* from *Aaronia AG* in order to avoid significant static offsets by earth’s magnetic field. The magnetic flux density is swept from negative to positive values and also backwards after magnetically saturating the sensor at $B_{\mathrm{sat}}=\mp 10\;\mathrm{mT}$ before each magnetic field sweep.

The static phase response $\varphi \left(B\right)=\arg(s_{21}\left(f_{0},B\right))$ shown in Figure 6a exhibits a significant dependence on the magnetic flux density *B*. Compared to the value $\varphi (B_{\mathrm{sat}})$ in magnetic saturation, the phase changes by up to about 7 rad $\left(\hat{\approx }400{}^{\circ }\right)$. Since the phase changes are significant especially in the ranges around B≈±0.2 mT, these regions are of particular interest for a later sensor operation (discussed below in Section 2.5). As a consequence of a slightly tilted magnetic anisotropy axis in the magnetic layer (Section 2.2) the phase responses are asymmetric and hysteretic such that the minimum values are reached just below or slightly above B=0, respectively [29].

The phase response can be expressed by the group delay $\tau_{g}\left(B\right)=-\partial \varphi \left(f,B\right)/\left(2\pi \;\partial f\right)$ or, analogously, by the group velocity $v_{g}\left(B\right)=L/\tau_{g}\left(B\right)$ as depicted in Figure 6b where the derivative of the phase response φ(f) was calculated in its linear regime (compare Figure 2a in [37]) around the sensor’s synchronous frequency ($f_{0}\pm 2\;\mathrm{MHz}$). With a value in the range of about 3340 m/s, the latter lies well between the theoretical bulk shear velocities $v_{\mathrm{sh}}=\sqrt{G/\rho }$ of quartz ( 4309 m/s) and the magnetostrictive FeCoSiB ( 2737 m/s) with *G* and ρ representing the shear modulus and the specific mass, respectively, of the individual material [37].

In addition to the phase changes φ(B), an analysis of the magnetic field dependent insertion loss $\mathrm{IL}(f_{0},B)$ reveals another significant dependence. As shown in Figure 6c, the insertion loss increases from the fundamental value of 20 dB in magnetic saturation to values of up to 39 dB and 41 dB, respectively, depending on the direction of the previously performed magnetic saturation. In fact, these high values occur in those ranges where the phase also changes significantly with the external magnetic field (compare Figure 6a). However, obviously, maximum losses only occur on one side with regard to B=0, namely after zero crossing. In contrast, each phase response has two steep slopes. This loss is related to the corresponding magnetic domain behavior as discussed in Section 2.2 and Section 2.4. The regime of increased electrical losses coincides with the occurrence of a multi-domain state and the shown energy transfer into the magnetic film. This leads to the dependence on the ambient magnetic field, due to the obvious hysteretic effects. These results clearly indicate an additional loss mechanism in the magnetic layer. Due to the general relation between losses and fluctuations the losses are of particular interest and are therefore characterized and discussed in detail further below.

### 2.5. Sensor Operation

When operating a SAW delay line sensor, the output signal is typically compared with its input signal to extract the desired information about the measurement signal. Although, in contrast to such open-loop systems, closed-loop or self-oscillating systems, respectively, are also common, an open-loop analysis of the sensor can be performed without any loss of generality [46].

Assuming an ideal oscillator signal, i.e., a sinusoidal signal without any fluctuations in amplitude and phase

(4) $$\begin{array}{c} P_{\mathrm{in}}\left(t\right)=P_{0}\sqrt{2}\;\cdot \;\cos\left(2\pi f_{0}t\right) \end{array}$$

to excite the sensor at its synchronous frequency $f_{0}$ with an input power of $P_{0}$, the sensor’s output signal can be described by

(5) $$\begin{array}{cc} P_{\mathrm{out}}\left(t\right) & =P_{0}\sqrt{2}\;{\left|s_{21}\left(f_{0},B\right)\right|}^{2}\;\cdot \;\cos\left(2\pi f_{0}t+\varphi \left(B\right)+\Delta \varphi \left(t\right)\right). \end{array}$$

The term Δφ(t) describes random phase fluctuations due to the sensor itself which are analyzed in detail in Section 3 and Section 4. In real sensor operation, a magnetic measurement signal $B_{x}\left(t\right)$ should generally be detected with high sensitivity. Therefore *B* is to be interpreted as the sum of $B_{x}\left(t\right)$ and an ambient static magnetic bias flux density $B_{\mathrm{bias}}$ which is generally applied for maximizing the sensitivity $S_{\mathrm{PM}}$. Thus, when neglecting any further changes and fluctuations of the signal’s amplitude, Equation (5) can be written as

(6) $$\begin{array}{cc} P_{\mathrm{out}}\left(t\right) & =P_{0}\sqrt{2}\;{\left|s_{21}\left(f_{0},B_{\mathrm{bias}}\right)\right|}^{2}\;\cdot \;\cos\left(2\pi f_{0}t+S_{\mathrm{PM}}\left(B_{\mathrm{bias}}\right)B_{x}\left(t\right)+\Delta \varphi \left(t\right)\right). \end{array}$$

According to Equation (1), the phase sensitivity $S_{\mathrm{PM}}$ can principally be obtained by the derivative of the phase response φ(B) (Figure 6a). However, it was found that this procedure leads to partially non-reproducible and incorrect results. In fact, a more precise distinction must be made. The slope of the phase response which corresponds with low insertion losses (Figure 6c) is typically unproblematic with regard to a numerical derivation. However, the slope that corresponds with significant insertion losses is often impaired by small phase jumps due to sudden and irreversible magnetic domain wall behavior (Section 2.3) which, in turn, will get even more pronounced when the derivative is calculated, thus, erroneously resulting in apparently high sensitivities. To overcome this issue, dynamic phase measurements for the determination of $S_{\mathrm{PM}}$ can be performed that are explained in Section 4.

### 2.6. Noise

The frequency dependent noise floor of a magnetic field sensor system is usually given by an amplitude spectral density in units of $T/\sqrt{\mathrm{Hz}}$, often also referred to as equivalent magnetic noise floor, detectivity, or limit of detection (LOD)

(7) $$\begin{array}{c} \mathrm{LOD}\left(f,B_{\mathrm{bias}},P_{0}\right)=\frac{\sqrt{S_{\varphi }}}{S_{\mathrm{PM}}}. \end{array}$$

It not only depends on the frequency *f* but also on the magnetic bias flux density $B_{\mathrm{bias}}$ and the sensor’s input power $P_{0}$. Although the phase sensitivity decreases above a certain cutoff frequency depending on the sensor’s geometry and its delay time [47], it is constant for frequencies below 10 kHz for the sensors under investigation. The term $S_{\varphi }$ describes the one-sided power spectral density (in units of ${\mathrm{rad}}^{2}/\mathrm{Hz}$) of the sensor’s random phase fluctuations Δφ(t) ([48], p. 22). Its logarithmic representation $10\log_{10}\left(S_{\varphi }\left(f\right)\right)$ is given in units of $\mathrm{dB}\;{\mathrm{rad}}^{2}/\mathrm{Hz}$. For historical reasons, the two-sided phase noise density spectrum L(f) defined as $L\left(f\right)=1/2\;S_{\varphi }\left(f\right)$ and usually given in units of dBc/Hz (dB below the carrier) is often used [49].

A useful model for describing the frequency dependence of a power spectral density of random phase fluctuations is the polynomial law

(8) $$\begin{array}{c} S_{\varphi }\left(f\right)=\sum_{i=-n}^{0}b_{i}f^{i} \end{array}$$

with usually n≤4. The exponents i=0 and i=−1 refer to white phase noise and 1/f flicker phase noise, respectively, which are usually the main noise processes in two-port components ([48], p. 23) like amplifiers [50]. However, under certain circumstances, magnetostrictively coated SAW delay line devices can also exhibit random walk of phase (i=−2).

In the following, it will be shown that a total of five different types of phase noise phenomena, namely

(1) $f^{0}$ white phase noise and
(2) $f^{-1}$ flicker phase noise

due to the SAW device itself and

(3) $f^{0}$ white phase noise,
(4) $f^{-1}$ flicker phase noise, and
(5) $f^{-2}$ random walk of phase

due to the additional magnetic material are observed depending on the sensor’s operating point.

## 3. Phase Noise in Magnetic Saturation

In this section the phase noise of SAW delay line elements both without any magnetostrictive coating as well as delay lines of which the sensitive layer is magnetically saturated by means of a permanent magnet field ($B=B_{\mathrm{sat}}\approx 10\;\mathrm{mT}$) is investigated. All phase noise measurements were performed at room temperature (T=290 K) utilizing an *FSWP* phase noise analyzer from *Rohde & Schwarz* while the SAW device itself is located inside an electrically, magnetically, and acoustically shielded measurement environment.

### 3.1. White Phase Noise

Energy equipartition in classical thermodynamics states that the thermal energy is $1/2\;k_{B}T$ per degree of freedom with $k_{B}\approx 1.38\times 10^{-23}\;J/K$ representing the Boltzmann constant ([51], pp. 264–266). For signals in the frequency range well below 6 THz (at room temperature) an overall noise energy of

(9) $$\begin{array}{c} N=k_{B}T=4\;\times \;10^{-21}\;J\;\hat{\approx }\;-174\frac{\mathrm{dBm}}{\mathrm{Hz}} \end{array}$$

is equally partitioned into the two degrees of freedom, i.e., amplitude and phase ([48], p. 42). Thus, for a sensor’s output signal with a power of $P_{0}{\left|s_{21}\right|}^{2}$ (Equation (6)), the white thermal phase noise is a factor of

(10) $$\begin{array}{c} L^{-1}=\frac{2P_{0}{\left|s_{21}\right|}^{2}}{N} \end{array}$$

below the carrier. This leads to a one-sided white phase noise power density ([48], p. 42) of

(11) $$\begin{array}{c} b_{0}=\frac{N}{P_{0}{\left|s_{21}\right|}^{2}}=\frac{\mathrm{IL}\;N}{P_{0}}=\frac{F\;N}{P_{0}} \end{array}$$

which is often expressed by the device’s noise figure $F=\mathrm{IL}=|s_{21}{|}^{-2}$ (linear representations of *F* and IL) and linearly decreases with higher input power levels $P_{0}$. Therefore, thermal phase noise, i.e., white phase noise, is referred to as *additive (phase) noise* ([48], p. 35).

**Figure 7:**
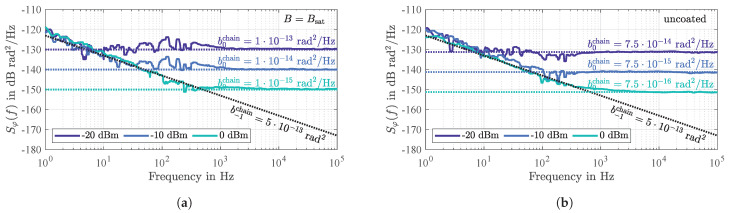
Measured power spectral densities $S_{\varphi }(f)$ of the random phase fluctuations Δφ(t) of the magnetostrictively coated sensor in magnetic saturation (**a**) and of an uncoated reference delay line (**b**). Both devices are measured for various input power levels $P_{0}$ and with an additional preamplifier. In agreement with Equation (11), the additive white phase noise decreases with $P_{0}$. Equal values for the parametric 1/*f* flicker phase noise are observed because both devices are located on the same chip.

The measurement results shown in Figure 7 were acquired with an additional amplifier *ZFL-1000LN+* from *Mini-Circuits* with a previously determined noise figure of $F_{\mathrm{AMP}}=1.875\;\hat{=}\;2.73\;\mathrm{dB}$ to amplify the sensor’s output signal. For such a chain of two devices, the overall white phase noise power density at the amplifier’s output can be determined by the adapted *Friis formula* ([48], p. 48)

(12) $$\begin{array}{c} b_{0}^{\mathrm{chain}}=\left(F+\frac{F_{\mathrm{AMP}}-1}{|s_{21}{|}^{2}}\right)\;\frac{N}{P_{0}}. \end{array}$$

For the SAW sensor under investigation previously introduced in Section 2, Figure 7a shows measured power spectral densities of random phase fluctuations for various input power levels $P_{0}$. As expected according to Equations (11) and (12), the white phase noise decreases by 10 dB each time the input power level is increased by 10 dB. Due to utilization of the additional amplifier, the measured coefficients $b_{0}^{\mathrm{chain}}$ contain additional phase noise of the amplifier. Calculating the sensor’s noise figure based on Equation (12) yields a value of F=21.3 dB which, within the measurement accuracy, agrees with the insertion loss of the sensor itself ( 20 dB) and additional losses of the connecting coaxial cables (previously determined to 1.2 dB). Thus, with regard to white phase noise, magnetostrictively coated SAW delay line sensors behave exactly as described by the existing noise theory. The white phase noise can be reduced by a higher input power level but increases with the insertion loss, regardless of the physical causes for the losses.

For comparison, a second series of measurements with the same measurement setup was performed for an uncoated reference delay line on the same chip as the previously measured magnetostrictively coated delay line (a photography of this chip can be found in [35]). The measurement results in Figure 7b show that the measured white phase noise is about 1.25 dB lower than for the sensitive delay line (Figure 7a) because the insertion loss of the uncoated device is lower by about the same amount. The reason for the slightly higher losses of the magnetically coated element are probably small defects in one of its two interdigital transducers (microscopic images of this imperfect transducer can be found in ([52], p. 362).

Losses due to eddy-currents always occur as soon as the coating material is electrically conductive. In [24] it was shown that the insertion losses significantly increase when a delay line is coated with thicker magnetic layers. If the thickness *z* of the magnetic layer is less than one skin depth δ (for the sensor under investigation δ≈1.4 μm ([53], p. 19), the power loss due to eddy-currents can be calculated by

(13) $$\begin{array}{c} P_{\mathrm{eddy}}=\frac{{\left(2\pi f_{0}\right)}^{2}{\hat{B}}_{0}^{2}Vz^{2}}{24\rho } \end{array}$$

where *V* is the volume of the magnetic layer, ρ is the magnetic material’s resistivity, and ${\hat{B}}_{0}$ is the amplitude of the magnetic flux density in the magnetic layer [54]. With ${\hat{B}}_{0}^{2}$ being proportional to the sensor’s input power $P_{0}$, Equation (13) can be written as

(14) $$\begin{array}{c} P_{\mathrm{eddy}}=\frac{{\left(2\pi f_{0}\right)}^{2}\gamma P_{0}Vz^{2}}{24\rho } \end{array}$$

with $\gamma ={\hat{B}}_{0}^{2}/P_{0}$. With this definition, the sensor’s additional insertion loss due to eddy-currents yield (linear representation)

(15) $$\begin{array}{c} {\mathrm{IL}}_{\mathrm{eddy}}=\frac{P_{0}}{P_{0}-P_{\mathrm{eddy}}}={\left(1-\frac{{\left(2\pi f_{0}\right)}^{2}\gamma Vz^{2}}{24\rho }\right)}^{-1}. \end{array}$$

Analytically, γ is not trivial to determine. However, based on time-resolved MOKE microscopy, the normalized amplitude of the magnetization ${\hat{M}}_{0}/M_{s}$ due to the surface acoustic wave and via the inverse magnetostrictive effect (Villari effect [55]) could be determined to values ${\hat{M}}_{0}/M_{s}<0.1$ for sensors with a magnetic layer thickness of z=200 nm at an input power of $P_{0}=10\;\mathrm{mW}\;\hat{=}\;10\;\mathrm{dBm}$. With ${\hat{M}}_{0}/M_{s}={\hat{B}}_{0}/B_{s}$ the coefficient γ can also be expressed as

(16) $$\begin{array}{c} \gamma =\frac{{\hat{B}}_{0}^{2}}{P_{0}}=\frac{{\left(\frac{{\hat{M}}_{0}}{M_{s}}B_{s}\right)}^{2}}{P_{0}} \end{array}$$

yielding a value of $\gamma <2.25T^{2}/W$ when assuming a saturation flux density of $B_{s}=1.5\;T$ [42] for the utilized (Fe${}_{90}$Co${}_{10}$)${}_{78}$Si${}_{12}$B${}_{10}$ alloy. With a volume of V=3.07 mm·3.92 mm·z (z=200 nm) and a resistivity of ρ=1.1 μΩm [56] for the amorphous FeCoSiB alloy, the calculated power loss and the insertion loss due to eddy-currents yield $P_{\mathrm{eddy}}<68\;\mu W$ (at $P_{0}=10\;\mathrm{mW}$) and ${\mathrm{IL}}_{\mathrm{eddy}}<1.0068\;\hat{=}\;0.03\;\mathrm{dB}$, respectively, which are neglectable values for such sensors. However, white phase noise due to eddy-current losses is not generally neglectable. For layer thicknesses of z=650 nm the insertion loss yields ${\mathrm{IL}}_{\mathrm{eddy}}=1\;\mathrm{dB}$ and further increases significantly for thicker layers, e.g., to ${\mathrm{IL}}_{\mathrm{eddy}}=8.2\;\mathrm{dB}$ for z=1 μm.

### 3.2. Flicker Phase Noise

Unlike frequency-independent (white) noise, the noise power of other noise phenomena is often confined at low frequencies. Although the power spectral densities describing these phenomena can have various spectral shapes, the most prominent example is the 1/f flicker noise which, with regard to the frequency, decreases with 10 dB/decade. Hence, 1/f noise is primarily disturbing in low-frequency applications. However, as soon as an additional carrier signal with a comparatively high amplitude is present, the noise also becomes noticeable around the carrier frequency, thus impairing the spectral components of a modulating signal ([48], p. 35). Besides a nonlinear mechanism, temporal fluctuations of the system properties can also cause the up-conversion of low-frequency noise ([48], pp. 44–45). An important characteristic of such *parametric amplitude and phase noise* is the independence from the carrier power.

For frequencies below the corner frequency

(17) $$\begin{array}{c} f_{c}=\frac{b_{-1}}{b_{0}} \end{array}$$

white phase noise $b_{0}$ becomes neglectable and the overall power spectral density $S_{\varphi }\left(f\right)$ is dominated by 1/f flicker phase noise described by the coefficient $b_{-1}$.

In a chain of several components, e.g., a delay line followed by an amplifier, the white phase noise of each component adds up according to Equation (12). On the contrary, the 1/f flicker phase noise at the output of such a chain

(18) $$\begin{array}{c} b_{-1}^{\mathrm{chain}}=b_{-1}+b_{-1}^{\mathrm{AMP}} \end{array}$$

is directly given by the sum of the individual 1/f flicker phase noise coefficients [48] (p. 49) (here $b_{-1}$ and $b_{-1}^{\mathrm{AMP}}$ represent the 1/f flicker phase noise components of the SAW device and of an additional amplifier).

In advance to the noise measurements of which the results are shown in Figure 7, the flicker phase noise coefficient of the utilized preamplifier *ZFL-1000LN+* from *Mini-Circuits* was determined to $b_{-1}^{\mathrm{AMP}}=6\times 10^{-14}\;{\mathrm{rad}}^{2}$. Thus, with measured flicker phase noise coefficients of $b_{-1}^{\mathrm{chain}}=5\times 10^{-13}\;{\mathrm{rad}}^{2}$ the SAW devices contribute a flicker phase noise of $b_{-1}=4.4\times 10^{-13}\;{\mathrm{rad}}^{2}$. Interestingly, both SAW devices, i.e., the magnetostrictively coated sensor as well as the uncoated reference delay line, show exactly the same flicker phase noise indicating that the additional magnetic layer does not contribute any further dominant flicker phase noise, at least if the sensitive layer is magnetically saturated. As characteristic for parametric noise, the flicker phase noise does not change with the input power.

**Figure 8:**
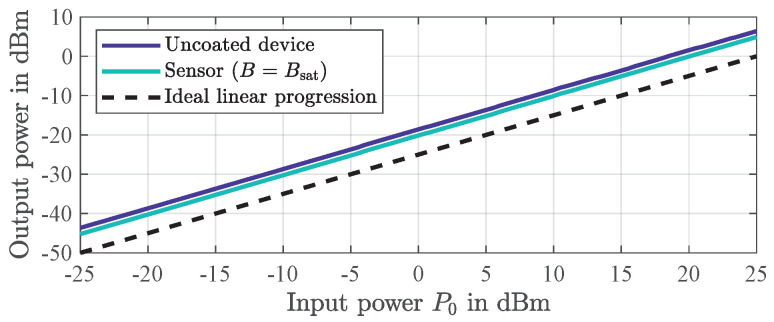
Measured output power as a function of input power $P_{0}$ of the two investigated SAW delay lines, i.e., the magnetostrictively uncoated device and the sensor in magnetic saturation. A strict linearity is revealed for both devices. The measurements were performed with a calibrated setup consisting of a signal generator *SMBV100A* and a signal and spectrum analyzer *FSV*, both from *Rohde & Schwarz*.

Figure 8 shows the output power of both devices as a function of the input power $P_{0}$, each revealing strict linearity. Thus, a nonlinear mechanism for the up-conversion of the 1/f flicker noise can be excluded. Instead, a quasi-linear parametric mechanism ([48], p. 45) due to fluctuating transmission characteristics of the delay line leads to noticeable noise around the carrier frequency. Various effects can cause these fluctuations whereas, so far, only a dominant flicker phase noise contribution of the magnetically saturated magnetostrictive layer can be excluded.

Previous studies identified IDT metalizations [57,58,59] and the piezoelectric substrate [58,59] as the major sources of flicker noise in SAW devices. Mobile impurities or defects in the substrate cause fluctuations in the local acoustic wave velocity [59], thus leading to random phase fluctuations. In addition, due to a very strong sensitivity to surface conditions, the surface acoustic wave velocity is modulated by gas molecules adsorbed onto the surface [58,59]. For example, as early as 1979, it was reported that the flicker noise depends on the cleanliness of the surface [60,61].

**Figure 9:**
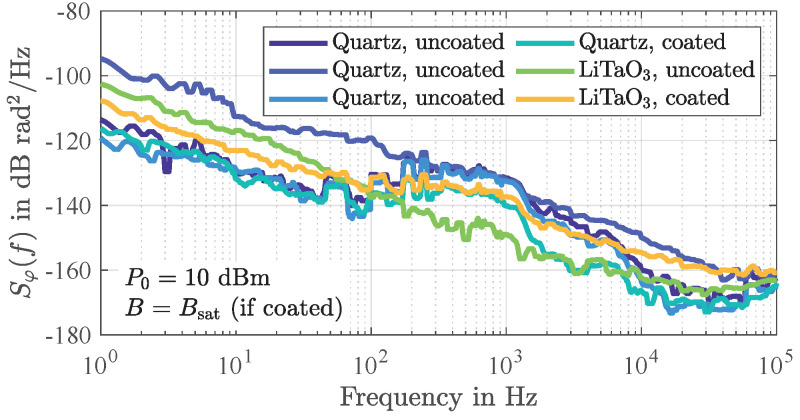
Measured power spectral densities of random phase fluctuations of several delay lines of basically the same design but from different wafers. The significant variance between the measured noise floors indicates differences in the purity of the materials that cause fluctuations in the local acoustic wave velocity. The labels *coated* and *uncoated* refer to the presence of a magnetostrictive layer. The measurements were performed at an electrical input power level of 10 dBm and without any additional amplifier.

Obviously, the elements of the sensor that are most critical in terms of fluctuations are those that are most involved in the acoustic wave generation, propagation, and re-conversion. Therefore, for the special case of surface acoustic Love wave devices, the additional guiding layer (here SiO${}_{2}$ with a thickness of 4.5 μm) is also expected to contribute to the overall flicker phase noise. Figure 9 shows the measured phase noise density spectra of several delay lines of basically the same design but from different wafers that are not only based on quartz but also on LiTaO${}_{3}$ substrates. Although the actual partial component responsible for the flicker phase noise is not apparent from this, the significant variance indicates differences in the purity of the materials. Apart from few studies on phase noise in SAW components mentioned above, most of which date back 30 to 40 years, surface acoustic Love wave elements in particular are still rarely investigated offering opportunities for future studies. However, as discussed in the following Section 4, in the special case of magnetostrictively coated SAW devices, additional phase noise of magnetic origin occurs to which the phase noise of bare devices is generally yet neglectable.

## 4. Phase Noise in Magnetic Operating Points

In this section, the phase noise, the phase sensitivity, and the insertion loss of the previously introduced magnetostrictively coated SAW delay line magnetic field sensor is analyzed for various practically relevant magnetic operating points.

### 4.1. Measurement Setup

**Figure 10:**
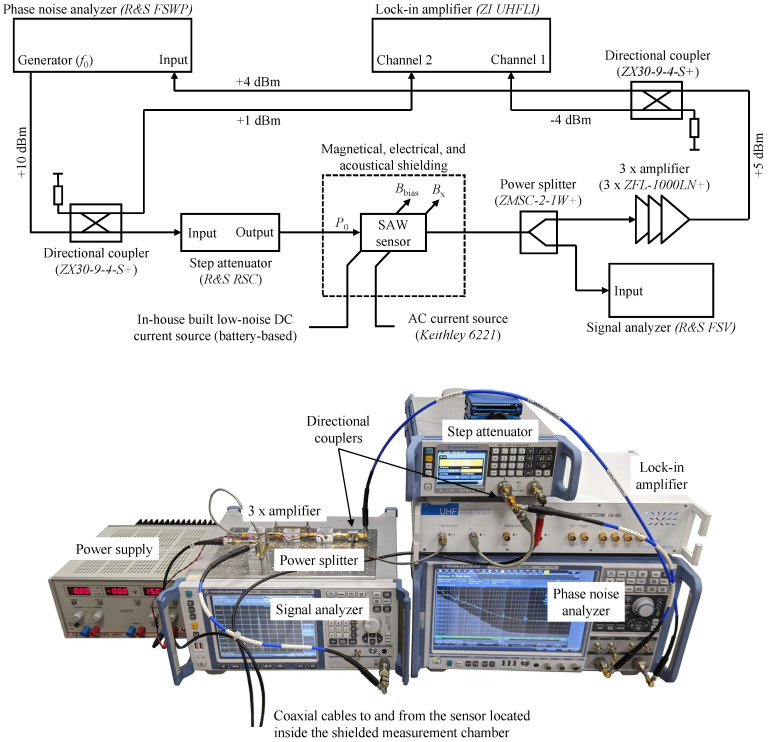
Block diagram (**top**) and photography (**bottom**) of the utilized system for the automatized measurement of the sensor’s phase sensitivity $S_{\mathrm{PM}}$, the phase noise $S_{\varphi }$, and the insertion loss IL as a function of both the sensor’s input power $P_{0}$ as well as the magnetic bias flux density $B_{\mathrm{bias}}$.

The automatized measurement system depicted in Figure 10 is designed to enable the measurement of the sensor’s phase sensitivity $S_{\mathrm{PM}}$, the phase noise $S_{\varphi }$, and the insertion loss IL as a function of both the sensor’s input power $P_{0}$ as well as the ambient magnetic bias flux density $B_{\mathrm{bias}}$.

As before, the SAW sensor itself is located in a magnetically (*ZG1* from *Aaronia AG*), electrically and acoustically shielded measuring chamber and is surrounded by two solenoids. These coils are used to generate the static magnetic bias flux density $B_{\mathrm{bias}}$ by means of an in-house built and battery-based low-noise direct current source and for the generation of the dynamic flux density $B_{x}\left(t\right)$ utilizing a commercial precision current source (*Keithley 6221*).

The internal generator of the phase noise analyzer (*FSWP* from *Rohde & Schwarz*) is used to excite the sensor at its synchronous frequency $f_{0}=144.8\;\mathrm{MHz}$. Because the output power of this integrated generator cannot be finely adjusted, a programmable step attenuator (*RSC* from *Rohde & Schwarz*) is utilized between the generator and the sensor which allows to alter the sensor’s input power $P_{0}$ in a wide range. In a separate analysis it was ensured that the flicker phase noise of the step attenuator can be neglected compared to the flicker phase noise of the SAW sensor under investigation. In fact, due to its design based on mechanical switches, the step attenuator’s flicker phase noise is even below the inherent flicker phase noise of the *FSWP* phase noise analyzer when configured to 100 cross-correlations.

After amplifying the sensor’s output signal utilizing three amplifiers connected in series (3 × *ZFL-1000LN+* from *Mini-Circuits*) with an overall gain of approx. 75 dB the signal is fed back to the input of the phase noise analyzer. The high gain is necessary in cases of low input power $P_{0}$ or high insertion loss IL, respectively, because the *FSWP* phase noise analyzer is not equipped with an internal preamplifier. On the other hand, at least one of these amplifiers is operated in compression if the sensor’s input power $P_{0}$ is relatively high or the sensor’s insertion loss is low. This leads to an increased noise figure $F_{\mathrm{AMP}}$ of the respective amplifier [50]. However, the flicker phase noise $b_{-1}^{\mathrm{AMP}}$ of these amplifiers does not increase when operated in compression, yielding an overall flicker phase noise of the amplifier chain of $b_{-1}^{\mathrm{chain}}=3\times b_{-1}^{\mathrm{AMP}}=1.8\times 10^{-13}\;{\mathrm{rad}}^{2}$.

In order to allow a determination of the sensor’s insertion loss, the sensor signal is analyzed by an additional and carefully calibrated signal analyzer (*FSV* from *Rohde & Schwarz*) after this signal is divided into two branches by means of a conventional 3 dB power splitter (*ZMSC-2-1W+* from *Mini-Circuits*). Utilizing two 9 dB directional couplers (*ZX30-9-4-S+* from *Mini-Circuits*) the amplified sensor output signal and the generator signal (phase reference) are fed into a lock-in amplifier (*UHFLI* from *Zurich Instruments*). It is operated as a phase demodulator whose output signal is used for the determination of the phase sensitivity $S_{\mathrm{PM}}$ (Equation (1)) by evaluating the amplitude spectrum of the demodulated phase signal φ(t) for calibrated amplitudes ${\hat{B}}_{x}=1\;\mu T$ of the dynamic flux density $B_{x}\left(t\right)={\hat{B}}_{x}\cos\left(2\pi f_{x}t\right)$ at a frequency of $f_{x}=10\;\mathrm{Hz}$. In addition, synchronously to noise measurements with the phase noise analyzer (while ${\hat{B}}_{x}=0$), the phase demodulator, i.e., the lock-in amplifier, is used to record the random phase fluctuations Δφ(t) (Equation (6)) as a time-domain signal.

The additional flicker phase noise of the passive components, i.e., the directional couplers and the power splitter, is usually as low as $b_{-1}^{\mathrm{passive}}<1\times 10^{-17}\;{\mathrm{rad}}^{2}$ and is thus negligible [62].

### 4.2. White Phase Noise

As mentioned in Section 3.1, a signal’s overall thermal noise floor of $N=k_{B}T$ corresponds with an *additive* white phase noise quantified by Equation (11). White phase noise $b_{0}$ decreases with higher signal power, i.e., for sensors with low insertion losses IL and high input powers levels $P_{0}$. Only the insertion losses are relevant here, regardless of the physical causes leading to the losses.

**Figure 11:**
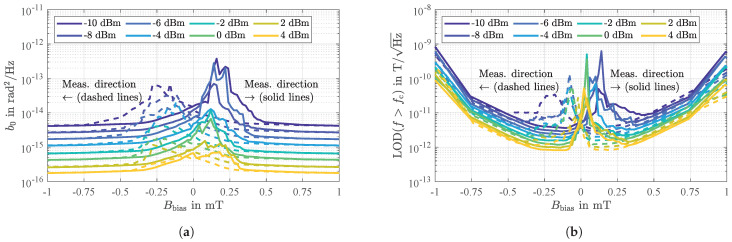
(**a**) Measured white phase noise *b*_0_ as a function of the ambient bias magnetic flux density *B*_bias_ and for various input power levels *P*_0_. The measurements were performed at the sensor’s synchronous frequency of *f*_0_ = 144.8 MHz and reveal the general trend of decreasing white phase noise for increasing input power levels. For magnetic flux densities around *B* = 0 additional magnetically induced insertion losses occur that lead to increased white phase noise. (**b**) Calculated limit of detection in the white noise regime according to Equation (19). The underlying phase sensitivity is shown in Figure 13c.

For the sensor under investigation, typical values for the white phase noise $b_{0}$ are depicted in Figure 11a as a function of the ambient bias magnetic flux density $B_{\mathrm{bias}}$ and for various input power levels $P_{0}$. For the sensor virtually being magnetically saturated (at $B_{\mathrm{bias}}=\pm 1\;\mathrm{mT}$) the white phase noise simply decreases by the same amount $P_{0}$ is increased. The same trend is also observed for small magnetic flux densities around $B_{\mathrm{bias}}=0$. However, in this region additional magnetically induced insertion losses occur (compare Figure 6c and Figure 13b that lead to increased white phase noise. This is consistant with the nucleation and presence of magnetic domain walls with the variation of $B_{\mathrm{bias}}$ discussed above.

According to Equation (7) and using Equations (11) and (9), the limit of detection above the corner frequency $f_{c}$ (Equation (17)), i.e., in the white noise regime

(19) $$\begin{array}{c} \mathrm{LOD}\left(f\right)\overset{f>f_{c}}{=}\frac{\sqrt{b_{0}}}{S_{\mathrm{PM}}}=\frac{1}{S_{\mathrm{PM}}}\sqrt{\frac{\mathrm{IL}\;k_{B}T}{P_{0}}}, \end{array}$$

directly scales with the phase sensitivity $S_{\mathrm{PM}}$ and further improves with lower insertion losses (linear representation of IL), lower temperatures, and higher input power levels. Based on Equation (19), values for the LOD in the white noise regime are displayed in Figure 11b where the underlying phase sensitivity $S_{\mathrm{PM}}$ will be discussed further below (Figure 13c). In principle, for input power levels above 0 dBm, white noise detectivities below 1 pT/$\sqrt{\mathrm{Hz}}$ can be reached. However, please note that such values are only reachable if the white noise corner frequency $f_{c}$ (Equation (17)) is below the cutoff frequency of the phase sensitivity [47].

### 4.3. Flicker Phase Noise

In Section 3.2 it was shown that a magnetically uncoated SAW delay line device contributes a flicker phase noise as low as $b_{-1}=4.4\times 10^{-13}\;{\mathrm{rad}}^{2}$. The same value is reached for the magnetically coated device when operated in magnetic saturation. However, sensors coated with a magnetostrictive layer that are operated outside magnetic saturation are impaired by additional low-frequency phase noise that depends on the magnetic bias flux density $B_{\mathrm{bias}}$.

**Figure 12:**
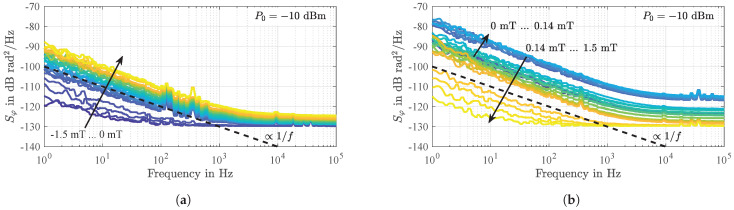
Measured power spectral densities of random phase fluctuations of the SAW delay line magnetic field sensor for increasing magnetic bias flux densities *B*_bias_ from −1.5 mT to 0 mT (**a**) and from 0 mT to 1.5 mT (**b**). Obviously, the phase noise significantly depends on the ambient magnetic flux density *B*_bias_. As for magnetically uncoated devices as well as for magnetically saturated sensors (Figure 7), in the low-frequency regime, a clear 1/*f* frequency dependence of the additionally and magnetically induced phase noise is revealed.

**Figure 13:**
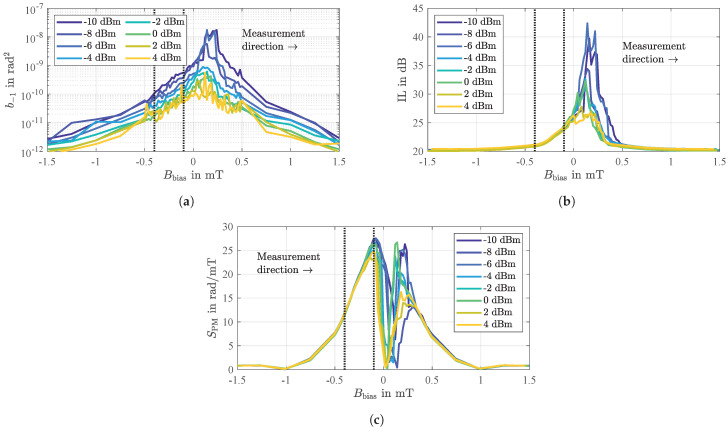
Measured flicker phase noise (**a**), insertion losses (**b**), and phase sensitivity (**c**) of the SAWmagnetic field delay line sensor as a function of the ambient magnetic bias flux density *B*_bias_ and for various input power levels *P*_0_. The sensor is operated preferably in a certain range (marked by dotted lines, here approximately between −0.4 mT and −0.1 mT) where the insertion losses and thus the flicker phase noise are comparatively low but the sensitivity is still high.

As shown in Figure 12 for the sensor being operated exemplary at an input power of $P_{0}=-10\;\mathrm{dBm}$, this phase noise decreases proportionally to 1/f so that it can also be referred to as flicker phase noise. It is also noticeable that this additional flicker phase noise increases with the ambient bias magnetic flux density $B_{\mathrm{bias}}$ up to a certain value (here 0.14 mT) and then decreases again. Noticeably the points of maximum flicker phase noise switch lower values of $B_{\mathrm{bias}}$ for higher input power levels $P_{0}$, indicating again a connection to magnetic domain wall occurrence. Extracting the flicker phase noise coefficients $b_{-1}$ as a function of $B_{\mathrm{bias}}$ results in a characteristic as shown in Figure 13a. Noticeably, maximum flicker phase noise coincides with the highest magnetically induced insertion losses (Figure 13b) and decreases when the insertion losses IL decrease, i.e., for $B_{\mathrm{bias}}$ approaching magnetic saturation and for higher input power levels $P_{0}$. In comparison to the previously determined flicker phase noise in magnetic saturation ($b_{-1}=4.4\times 10^{-13}\;{\mathrm{rad}}^{2}$, Figure 7a) highest insertion losses correspond with an increase in flicker phase noise power by more than a factor of 40,000 or 46 dB, respectively. The regime of highest noise coincides with the regime of high magnetic energy transfer into magnetic domain walls (Figure 5), indicating a connection to magnetic domain wall processes. In investigations on magnetoresistive sensors an identical behavior could be observed in the past [63,64]. These sensors also show the largest noise for operating points of maximum sensitivity which was attributed to random fluctuations of the magnetization due to magnetic domain wall movements and rotations [64,65].

Apparently, when also considering the phase sensitivity $S_{\mathrm{PM}}$ (Figure 13c), there is a certain magnetically stable range (marked by dotted lines and for this sensor approximately between −0.4 mT and −0.1 mT) in which the sensor is to be operated preferably, i.e., where the insertion losses and thus the flicker phase noise are comparatively low but the sensitivity is still relatively high. This region coincides with the low domain wall density regime (Figure 3) with low magnetic losses discussed in detail in Section 2.4. Note that these measurements were performed for increasing magnetic bias flux densities after saturation in negative direction. An inverted measurement started at positive magnetic saturation virtually yields identical results only with reversed signs (compare e.g., Figure 6c), again coinciding with the bias field asymmetry of magnetic domain behavior and density (Figure 4).

Due to the significant relation to the additional magnetic insertion losses it is obvious to describe the magnetically induced phase noise using the fluctuation-dissipation theorem. Based on that theorem, a power spectral density of random fluctuations of the magnetization

(20) $$\begin{array}{c} S_{M}\left(f\right)=\frac{4k_{B}T}{2\pi f\;V}\frac{\mu_{r,\mathrm{eff}}^{\prime \prime }}{\mu_{0}} \end{array}$$

with the physical dimension ${\left(A/m\right)}^{2}/\mathrm{Hz}$ can be derived [66,67]. It can be referred to as flicker magnetization noise since the power spectral density $S_{M}\left(f\right)$ decreases with 1/f. This expression is typically given as a function of the imaginary part $\mu_{r}^{\prime \prime }$ of the magnetic material’s complex permeability $\mu_{r}=\mu_{r}^{\prime }-j\mu_{r}^{\prime \prime }$. In general, however, $\mu_{r}^{\prime \prime }$ is also used to account for other losses, in particular eddy-current losses, which in turn do not correspond with flicker noise but with frequency-independent white noise [68,69]. Therefore, an effective complex permeability $\mu_{r,\mathrm{eff}}=\mu_{r}^{\prime }-j\mu_{r,\mathrm{eff}}^{\prime \prime }$ is introduced to cover only for magnetic hysteresis losses corresponding with 1/f flicker noise. Furthermore, $\mu_{0}$ and *V* denote the vacuum permeability and the volume of the magnetic material. Note that *f* denotes the Fourier frequency which is not equal to the delay line sensor’s synchronous frequency $f_{0}$.

With the magnetic susceptibility $\chi =\partial M/\partial H=\mu_{r}^{\prime }-1$ as the relationship between the magnetic field strength *H* and the magnetization *M*, the relation between phase changes and magnetization changes

(21) $$\begin{array}{cc} \frac{\partial \varphi }{\partial M} & =\frac{\partial \varphi }{\partial H}\;\cdot \;\frac{\partial H}{\partial M}=\frac{\partial \varphi }{\partial \mu_{0}H}\;\cdot \;\frac{\mu_{0}}{\chi }=S_{\mathrm{PM}}\;\cdot \;\frac{\mu_{0}}{\mu_{r}^{\prime }-1} \end{array}$$

can be expressed as a function of the phase sensitivity $S_{\mathrm{PM}}$ (Equation (1)). Thus, the flicker phase noise coefficient yields

(22) $$\begin{array}{cc} b_{-1} & =f\;\cdot \;{\left(\frac{\partial \varphi }{\partial M}\right)}^{2}\;\cdot \;S_{M}\left(f\right)=S_{\mathrm{PM}}^{2}\;\cdot \;\frac{4k_{B}T}{2\pi \;V}\frac{\mu_{0}\mu_{r,\mathrm{eff}}^{\prime \prime }}{{\left(\mu_{r}^{\prime }\right)}^{2}} \end{array}$$

when assuming that $\mu_{r}^{\prime }\gg 1$, which is true for our soft magnetic material (Figure 2). Note that for the discussion here, the domain wall susceptibility or the easy axis magnetic field behavior might be the relevant figure of merit. Equivalently, for the low-frequency flicker noise regime below the corner frequency $f_{c}$ (Equation (17)) the power spectral density of random phase fluctuations is given by

(23) $$\begin{array}{cc} S_{\varphi }\left(f\right) & \overset{f<f_{c}}{=}\frac{b_{-1}}{f}=S_{\mathrm{PM}}^{2}\;\cdot \;\frac{4k_{B}T}{2\pi f\;V}\frac{\mu_{0}\mu_{r,\mathrm{eff}}^{\prime \prime }}{{\left(\mu_{r}^{\prime }\right)}^{2}}. \end{array}$$

According to Equation (7), the limit of detection in the flicker noise regime then yields

(24) $$\begin{array}{c} \mathrm{LOD}\left(f\right)\overset{f<f_{c}}{=}\frac{\sqrt{b_{-1}f^{-1}}}{S_{\mathrm{PM}}}=\sqrt{\frac{2k_{B}T}{V\;\pi f}\;\frac{\mu_{0}\mu_{r,\mathrm{eff}}^{\prime \prime }}{{\left(\mu_{r}^{\prime }\right)}^{2}}}\approx \underset{\mathrm{for}\;V=2.41\;\times \;10^{-12}\;m^{3}}{\underbrace{\frac{36.5\mathrm{nT}}{\sqrt{f}}\;\cdot \;\sqrt{\frac{\mu_{r,\mathrm{eff}}^{\prime \prime }}{{\left(\mu_{r}^{\prime }\right)}^{2}}}}} \end{array}$$

which, on the contrary to the LOD in the white noise regime (Equation (19)), no longer depends on the phase sensitivity $S_{\mathrm{PM}}$. In fact, the LOD in the flicker noise regime is mainly determined by the complex-valued permeability of the magnetic material. Recently, we have confirmed this result in two studies. An investigation on SAW delay lines with magnetic layers of different thicknesses has shown that, although the sensitivity increases significantly with thicker layers, the LOD in the flicker noise regime remains constant due to increasing magnetic losses [24]. A comparison between the operation of such a sensor in the fundamental and the first higher Love wave mode also showed that, although both sensitivities significantly differ, similar limits of detection in the flicker noise regime resulted [70]. Another recently published investigation on ferrite flux concentrators utilized with diamond magnetometers [69] also comes to the same conclusion that the relative loss factor $\mu_{r,\mathrm{eff}}^{\prime \prime }/{\mu_{r}^{\prime }}^{2}$ must be limited in order to minimize hysteresis noise.

**Figure 14:**
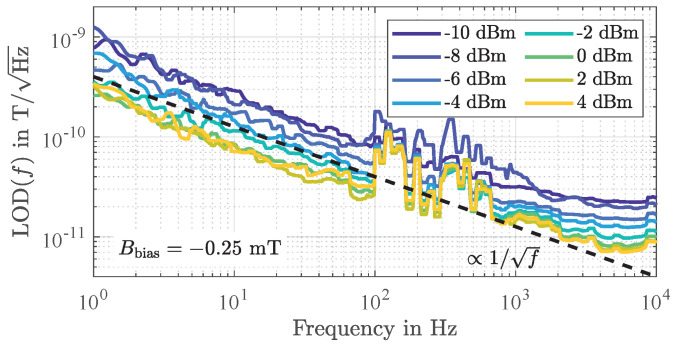
Measured equivalent magnetic noise floors for various input power levels $P_{0}$ at an ambient magnetic bias flux density of $B_{\mathrm{bias}}=-0.25\;\mathrm{mT}$ (after a negative magnetic saturation). The $1/\sqrt{f}$ dependency confirms Equation (24), i.e., magnetic hysteresis losses dominate under these operating conditions.

For the sensor under investigation operated at an ambient magnetic bias flux density of $B_{\mathrm{bias}}=-0.25\;\mathrm{mT}$, detectivities as depicted in Figure 14 were measured for various input power levels $P_{0}$. In accordance with Equation (24), all measured equivalent magnetic noise spectra improve with $1/\sqrt{f}$ confirming that magnetic hysteresis losses, i.e., random fluctuations of the magnetization, dominate under these operating conditions. However, because the flicker phase noise depends on $P_{0}$ (Figure 13a), the LOD also improves with increasing input power levels up to a value of about 70 pT/$\sqrt{\mathrm{Hz}}$ at an exemplary frequency of 10 Hz for optimum input power levels between 0 dBm and 4 dBm. Thus, the magnetic losses represented by $\mu_{r,\mathrm{eff}}^{\prime \prime }$ depend on the input power level $P_{0}$.

**Figure 15:**
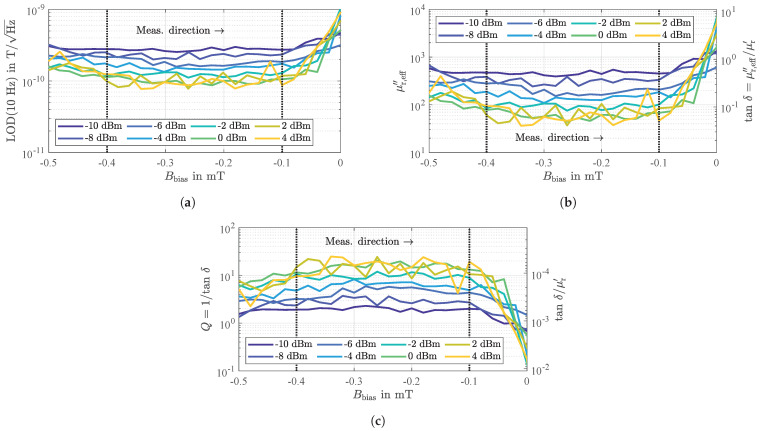
Measured limit of detection at an exemplary frequency of 10 Hz (**a**) and determined magnetic key figures (**b**,**c**) on the basis of Equation (25). On one hand, the results confirm the detectivity’s independence of the phase sensitivity and constant magnetic properties in a wide range of magnetic bias flux densities (dotted lines). On the other hand, a significant dependence of the magnetic properties, and thus the sensor performance, on the input power level is revealed. The underlying measurements were performed after a previously performed magnetic saturation in negative direction.

The previously discussed measurement results (Figure 13) also revealed a significant dependence of the flicker phase noise and the phase sensitivity on the ambient magnetic bias flux density. Nevertheless, as shown in Figure 15a, the LOD remains virtually constant over a comparatively large range with respect to $B_{\mathrm{bias}}$ between −0.4 mT and −0.1 mT (marked by dotted lines), thus confirming the independence of the phase sensitivity $S_{\mathrm{PM}}$. In contrast, the dependence on the input power is significant, indicating again a dependence of the magnetic properties on $P_{0}$.

Based on the measurement results and Equation (22) the imaginary part of the magnetic material’s effective complex permeability

(25) $$\begin{array}{c} \mu_{r,\mathrm{eff}}^{\prime \prime }=\frac{b_{-1}V\pi }{2k_{B}T\mu_{0}}\;\cdot \;{\left(\frac{\mu_{r}^{\prime }}{S_{\mathrm{PM}}}\right)}^{2} \end{array}$$

can be determined. Depending on the input power of the sensor, $\mu_{r,\mathrm{eff}}^{\prime \prime }$ is in the range between about 500 ($P_{0}=-10\;\mathrm{dBm}$) and 50 ($P_{0}=4\;\mathrm{dBm}$) corresponding with magnetic loss factors $\tan\delta =\mu_{r,\mathrm{eff}}^{\prime \prime }/\mu_{r}^{\prime }$ ([71], p. 33) of about 0.6 and 0.06 (Figure 15b). Because the ferromagnetic resonance frequency of FeCoSiB thin films is typically above 1 GHz rather low losses in the frequency range around 150 MHz would have been expected [72,73] assuming simple Landau-Lifshitz-Gilbert (LLG) resonance behavior [74]. Yet, for similar amorphous magnetic films [75] and FeCoSiB films of similar thicknesses [76], domain wall resonance effects in the lower 100 MHz regime have been reported and the losses were directly connected to magnetic domain wall resonances. Eddy-current effects should not play a role in the magnetic domain wall losses [77], only internal damping is of relevance. If one considers the magnetic quality factor Q=1/tanδ with values of up to about 25 or the relative magnetic loss factor $\tan\delta /\mu_{r}^{\prime }$ with values slightly below $10^{-4}$ (each for $P_{0}=4\;\mathrm{dBm}$) comparable values can be found in literature [66,78]. In fact, a similar value for the relative magnetic loss factor of $1.6\times 10^{-4}$ has recently been found for a resonant magnetic field sensor with a magnetostrictive thin-film of the same alloy utilized here [79].

### 4.4. Random Walk of Phase

So far, the sensor’s phase noise was primarily considered as a function of the frequency and the ambient magnetic bias flux density. However, measurements at selected power levels up to 4 dBm have already shown a significant influence of the sensor’s electrical input power on the phase noise performance.

**Figure 16:**
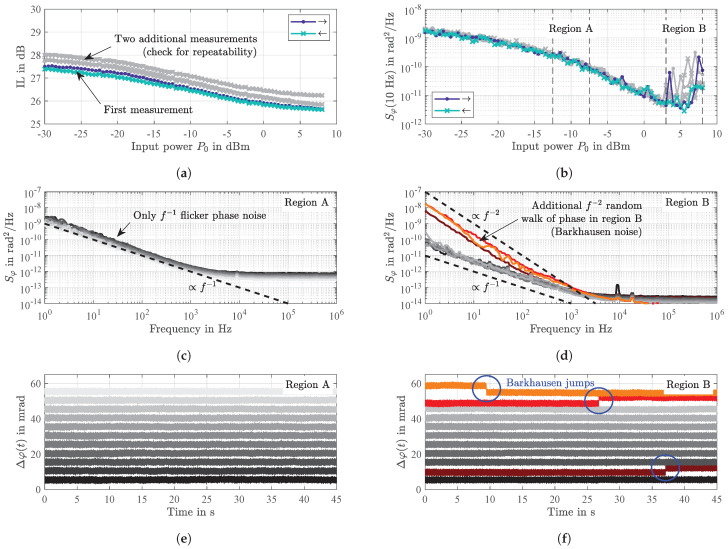
Measured loss and phase noise characteristics as a function of the sensor’s electrical input power *P*_0_ at a constant ambient magnetic bias flux density *B*_bias_ = 0 (after negative saturation). A direct relation between (hysteresis) losses (**a**) and phase noise at an exemplary frequency of 10 Hz (**b**) is revealed for input power levels *P*_0_ approx. below 4 dBm. In this regime, i.e., in *region A*, the power spectral densities of random phase fluctuations progress proportionally to 1/*f* (**c**). At higher power levels, i.e., in *region B*, random walk of phase (1/*f*^2^) occurs (**d**) that is caused by *Barkhausen jumps* (**f**) that do not occur at lower power levels (e). Please note the artificial phase offsets for clearer illustration in (**e**,**f**).

The results of a series of measurements as a function of the sensor’s input power $P_{0}$ at a constant ambient magnetic bias flux density of $B_{\mathrm{bias}}=0$ (after magnetically saturating the sensor in negative direction) are depicted in Figure 16. As observed before, the insertion losses decrease with higher $P_{0}$ by about 1.8 dB in the considered range from −30 dBm to 8 dBm (Figure 16a). Decreasing $P_{0}$ again virtually results in the same values. Furthermore, two additional measurements performed (gray) confirm the repeatability of this experiment. The only differences are marginally shifted curves due to different magnetic domain configurations after magnetically saturating the sensor. Although the losses decrease only moderately with higher $P_{0}$, a significant reduction of the phase noise (here exemplary at a frequency of 10 Hz) by a factor of about 640 is observed over a wide range (Figure 16b). For all power levels approximately below 4 dBm the power spectral densities of random phase fluctuations progress strictly proportional to 1/f as shown exemplary in Figure 16c for $P_{0}$ increasing from −12.5 dBm to −7.5 dBm, referred to as *region A*. These results again confirm the previously discussed dominance of 1/f flicker phase noise due to random fluctuations of the magnetization directly related to magnetic hysteresis losses.

However, if $P_{0}$ is further increased, the phase noise in all three series of measurements increases again, partly significantly (Figure 16b), although the losses continue to decrease slightly or stagnate at a constant level (Figure 16a) indicating the occurrence of an additional mechanism. This regime is referred to as *region B* and occurs approximately above 3 dBm for the sensor under investigation. A consideration of the associated power spectral densities of random phase fluctuations (Figure 16d) reveals that this increased phase noise corresponds with slopes of $1/f^{2}$ (highlighted by reddish colors), referred to as random walk of phase ([48], p. 23). In contrast to region A (Figure 16e), the corresponding time signals in region B show jumps, also highlighted by reddish colors (Figure 16e). From literature it is well-known that stochastic changes of the size of magnetic domains correspond with $1/f^{2}$ slopes in the associated power spectral densities ([80], p. 281). Therefore, the random walk of phase is caused by so-called *Barkhausen jumps*. In the following we prove that the magnetic fluctuations are related to hopping of magnetic domain walls.

**Figure 17:**
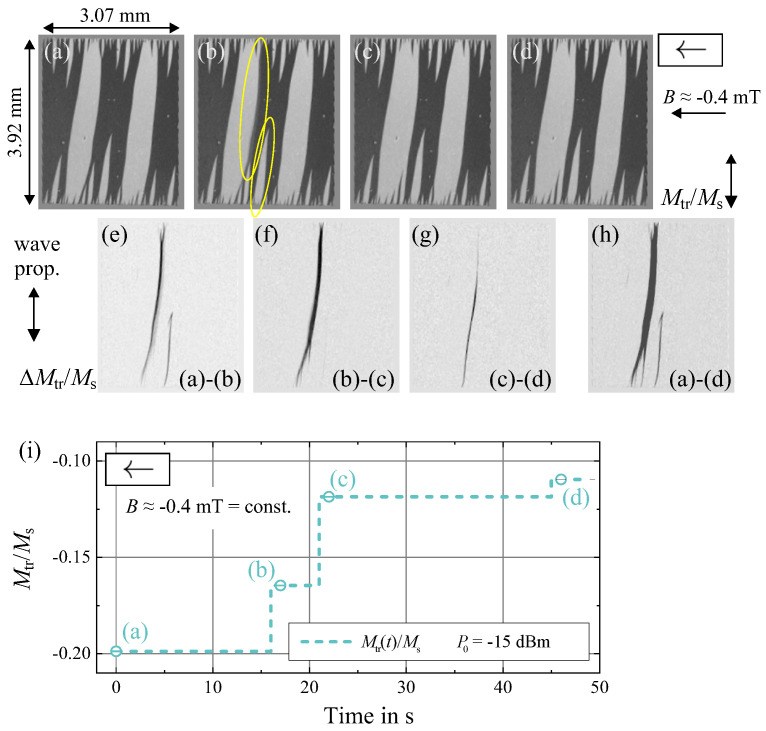
Magnetic domain observations with a constant magnetic bias field of B≈−0.4 mT. (**a**–**d**) Magnetic domain structure over time with an input power of −15 dBm. (**e**–**h**) Differential domain images displaying the alteration in the magnetic domain states over time. (**i**) Change of the transversal magnetization component $M_{\mathrm{tr}}/M_{s}$ with time. The positions of high domain activity are indicated in (**b**).

The assumption of low-frequency domain wall switching events is proven by in-situ magnetic domain observations. In Figure 5 we have shown that the magnetic switching process is altered with the electrical input power. The sporadic reorientation of magnetic domains without an alteration of the magnetic field is demonstrated in Figure 17. In addition to the magnetic domain states in Figure 17a–d the difference in the magnetic domain states over time is displayed in Figure 17e–h. The magnetic domains reorient across several seconds even for the given small input power. Magnetic domain walls move to a more stable state with time, where the probability of occurrence of magnetic domain reorientation increases with the electrical input power. Therefore, for each domain wall jump, the magnetization component $M_{\mathrm{tr}}/M_{s}$ increases. For the shown example, the overall process is mostly limited to two domain walls. The reorientation process in the negative bias field is a direct consequence of the slight misorientation of the magnetic anisotropy axis in the system. It should further be noted that the probability of domain switching events will also depend on the reverse magnetic bias field value, increasing drastically approaching the domain reorientation field discussed in Section 2.3. Electrically induced changes of the magnetic structure with zero field are also visible from the transverse magnetization curve data (Figure 5a), where a reduction of transverse remanence $B_{r}$ becomes already visible with $P_{0}=-10\;\mathrm{dBm}$.

## 5. Conclusions

In this paper, the noise behavior of SAW delay line magnetic field sensors coated with a thin-film of magnetostrictive material is investigated by means of extensive measurements, the results of which were used to describe the noise analytically. Such sensors utilize the magnetoelastic ΔE effect that leads to a magnetically induced alteration of the surface acoustic wave’s propagation velocity. Electroacoustic transducers at the sensor’s input and output port are utilized to generate the SAW and to provide an electrical signal whose phase contains the information about the magnetic field strength. Due to various sensor-intrinsic phenomena the output signal is impaired by phase noise that limits the detectivity.

Besides a discussion of the sensor’s electrical properties around the synchronous frequency of 144.8 MHz, insights into its magnetization behavior are given based on the magnetic domain behavior obtained by means of magnetooptical Kerr effect microscopy and magnetometry. An asymmetric domain behavior is revealed in which, coming from magnetic saturation, spike domains develop at the edges. After remanence, the spike domains grow and further on penetrate the whole sample, forming large magnetic domains that are directly linked with a magnetic energy transfer from a generated surface acoustic wave into the magnetic layer. These asymmetric and bias field dependent losses are also reflected in the electrical transmission properties of the sensor.

With regard to the spectral shape, it is revealed that SAW delay line magnetic field sensors exhibit three different types of phase noise, each with various causes.

Fundamental $f^{0}$ thermal phase noise, i.e., white phase noise, is directly linked to the sensor’s insertion loss, regardless of the physical causes for the loss. Typically, the insertion loss results from the static losses of the delay line structure and from the above mentioned dynamic hysteresis losses in the magnetic layer. In contrast, eddy-current losses are negligible in this frequency range and for such thin magnetic layers. White noise is additive noise that decreases with increasing signal power. For an optimal LOD in the white noise regime a high input power should be chosen at a magnetic operating point where magnetic losses are as low as possible while maintaining high phase sensitivity. In principle, the LOD in the white noise region can be reduced arbitrarily by increasing the input power. However, since the corner frequency of the white phase noise must remain below the cutoff frequency of the phase sensitivity, achievable values for the LOD in the white noise regime are typically in the range around 1 pT/$\sqrt{\mathrm{Hz}}$.

Every SAW delay line device exhibits fundamental $f^{-1}$ flicker phase noise due to the delay line structure itself, originating e.g., from mobile impurities or defects in the substrate and the guiding layer that cause fluctuations in the local acoustic wave velocity. This quasi-linear parametric mechanism is characterized by the fact that $f^{-1}$ flicker phase noise is generally independent of the sensor’s input power. It was found that magnetostrictively coated delay lines operated in magnetic saturation exhibit exactly the same $f^{-1}$ flicker phase noise as bare devices, i.e., delay lines without any additional magnetostrictive layer. Outside magnetic saturation, however, the $f^{-1}$ flicker phase noise significantly increases depending on the ambient magnetic bias field by more than 40 dB where maximum flicker phase noise coincides with highest magnetically induced insertion losses, i.e., with the occurrence of magnetic domain walls. Therefore, such sensors are preferably operated in magnetic operating points with low magnetic losses, i.e., in the low domain wall density regime. In this regime, and in agreement with calculations based on the fluctuation-dissipation theorem, measurements confirmed the independence of the LOD from the phase sensitivity. In contrast to the white noise regime, the LOD in the flicker noise regime cannot be improved by increasing the phase sensitivity. Instead, the magnetic losses must be limited in order to minimize hysteresis loss. Although flicker phase noise is inherently independent of the sensor’s input power, a significant dependence of flicker phase noise on the input power was found. This is due to the fact that the magnetic losses are power dependent, i.e., the magnetic losses decrease with higher input power levels. For optimal power levels in the range between 0 dBm and 4 dBm and at an exemplary frequency of 10 Hz, an LOD of 70 pT/$\sqrt{\mathrm{Hz}}$ could be achieved that corresponds with a relative magnetic loss factor of about $10^{-4}$.

If the electrical input power of the sensor is increased further, the phase noise power spectral density no longer shows a strict $f^{-1}$ slope. Instead, dominant $f^{-2}$ random walk of phase noise occurs. It was found that this random walk of phase is directly linked to sporadic reorientations of magnetic domains without an alteration of the magnetic field, i.e., Barkhausen jumps. Therefore, for best performance of such sensors, the electrical input power should generally be chosen as high as possible, but below the power range in which domain network reorientation processes occur.

## Data Availability

Not applicable.
